# Dynamic modulation of Poincaré beams

**DOI:** 10.1038/s41598-017-07437-9

**Published:** 2017-08-14

**Authors:** C. Alpmann, C. Schlickriede, E. Otte, C. Denz

**Affiliations:** University of Muenster, Institute of Applied Physics, Corrensstr. 2/4, 48149 Muenster, Germany

## Abstract

Generation of complex Poincaré beams is enabled by amplitude and phase modulation accompanied by simultaneous spatially polarization structuring. A holographic system to tailor complex light fields and optical angular momentum structures forecasts promising applications in quantum communication and optical trapping. Experimental results are presented together with simulations of complex Poincaré beams embedding different types of polarization singularities. Additionally, parameters of the dynamic polarization modulation system are discussed and analyzed to demonstrate the enormous capability of the method.

## Introduction

Polarization is one of the most fundamental yet fascinating properties of transverse waves, as light waves, gravitational waves, and sound waves in solids. For polarization studies, the polarization of light waves is a representative being accessible easily to experimental demonstrations, and allows characterizing as well as manipulating polarization in all its features. While homogeneously distributed states of polarization have been investigated comprehensively, recent research focuses on spatially inhomogeneous distributions of polarization, i.e. the coexistence of several states of polarization in complex transverse light fields. These so-called Poincaré beams^1, 2^ include combinations of linear states of polarization known as vector beams^3^ as well as elliptical and circular polarization states in spatial structures. Moreover, Poincaré beams are strongly connected to singular optics^4^ due to the appearance of different types of vectorial singularities within light fields revealing structured polarization^5^. Different techniques have been proposed to generate inhomogeneous distributions of states of polarization as e.g. the superposition of spatial modes^6, 7^, the application of polarization holograms^8^, q-plates^9, 10^, or plasmonic metasurfaces^11^ and dynamic techniques using spatial light modulators^12–16^ and digital micro-mirror devices^17^. Recently the combination of polarization modulation with amplitude or phase modulation has been investigated^18–21^ more intensively. However, a dynamic technique to modulate extended polarization structures is essential for investigations of advanced polarization singularity formations.

In this paper, we present the experimental realization of dynamically modulated Poincaré beams. Primarily, the polarization of Laguerre-Gaussian (LG) beams is designed according to the structure of its amplitude distribution. Furthermore, different combinations of spin and orbital angular momentum are modulated in a helical LG beam. Finally, polarization singular structures are enabled which is demonstrated by a spiraling Poincaré beam containing various kinds of polarization singularities. All results are experimentally implemented by a dynamic modulation technique which is discussed within the method section. It is realized by a single spatial light modulator which is in combination with a pair of wave plates capable of generating all kinds of fully-polarized polarization states. As we use a pixelated display, various combinations of coexisting states of polarization become possible in a spatially highly resolved pattern. To verify high modulation quality of extended polarization patterns, a spatially resolved measurement of Stokes parameters (see method sections) is employed^22^.

The advantage of our approach is the automatic alignment of horizontal and vertical polarization components due to the dynamic modulation technique. Opposed to previous studies^23–25^, an explicit superposition and alignment of orthogonally polarized modes is not necessary. This avoids propagation instabilities of (singular) polarization fields due to misalignment and gives the opportunity to investigate the longitudinal evolution of such structures.

## Results

**Figure 1:**
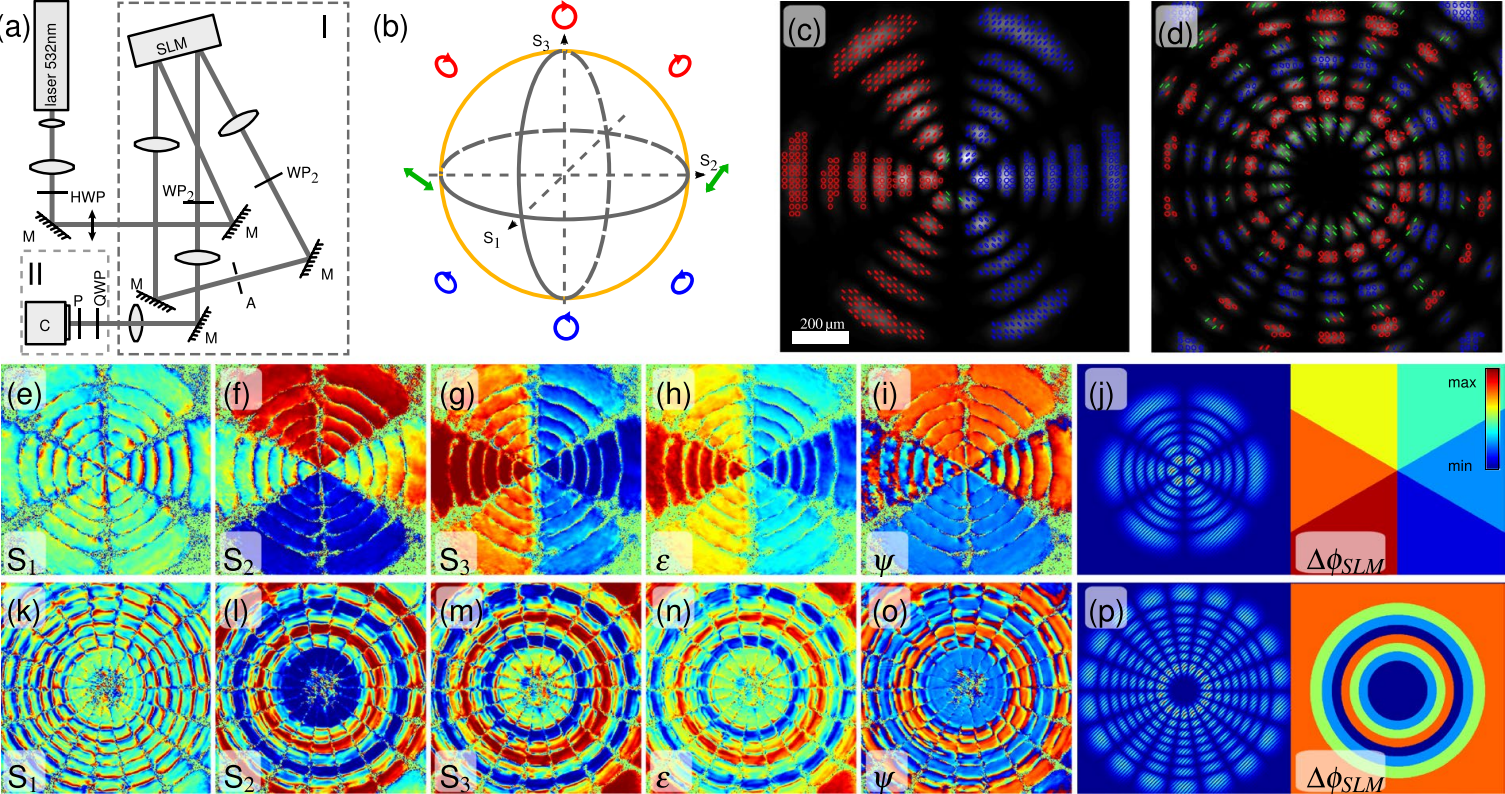
Polarization structured LG beams. (**a**) Experimental setup: polarization measurement system (II) and DPMS combined with phase and amplitude modulation (I). C: camera, (H/Q)WP: (half/quarter) wave plate, M: mirror, P: polarizer. (**b**) Schematic of the subspace (*θ*_1_ = −45°, *θ*_2_ = 90°). (**c**) ${{\rm{LG}}}_{\mathrm{5,3}}^{e}$ with azimuthally and (**d**) ${{\rm{LG}}}_{\mathrm{7,9}}^{o}$ with radially modulated polarization structure. Polarization states are depicted by red or blue ellipses/circles indicating right and left elliptical/circular polarization, respectively. Linear polarization states are shown by green lines. Stokes parameters S_1,2,3_ ∈ [−1, 1] (**e**–**g**,**k**–**m**) as well as the ellipticity *ε* ∈ [−1, 1] (**h**,**n**) and the orientation *ψ* ∈ [−*π*/2, *π*/2] (**i**,**o**) of polarization ellipses are given. Corresponding side-by-side holograms are shown in (**j**,**p**).

To access simultaneous holographic modulation of amplitude, phase as well as polarization, we employ a two step method experimentally implemented by a split-screen configuration^26^ of a parallel aligned nematic liquid crystal spatial light modulator (SLM) as depicted in Fig. 1(a). In the first step, a combined amplitude and phase encoding technique^27, 28^ allows the realization of e.g. higher order laser modes as Hermite-, Laguerre- or Ince-Gaussian beams. However, the method is not restricted to these modes but enables to modulate arbitrary light fields. In the second step, a dynamic polarization modulation system (DPMS) is implemented using the second half of the SLM (see Methods below) to realize spatially polarization structured Poincaré beams. Furthermore, our system enables to demonstrate orbital and spin angular momentum coupling as well as to create and study polarization singularities as V-points, C-points, L-lines and C-lines as discussed in the following.

### LG beams exhibiting discrete spatial polarization structures

Complex light fields is a common term in modern optics for a variety of light fields tailored spatially in one or various parameters. In this context, generation of extra-cavity higher order light modes is an established field enabled by employment of liquid crystal spatial light modulators. We modulate such higher order light modes which are not only structured in amplitude and phase, but also in polarization. Therefore, we generate higher order Laguerre-Gaussian (LG_*n*,*l*_) modes and simultaneously modulate polarization states spatially^29^. In Fig. 1 two examples are given: An even ${{\rm{LG}}}_{5,3}^{e}$ beam with azimuthal variation of six different polarization states shown in (c, e-j), and an odd ${{\rm{L}}{\rm{G}}}_{7,9}^{o}$ beam with radial variation of four different polarization states shown in (d, k-p). To verify modulation quality, the state of polarization is determined by spatial Stokes parameter measurements (s. method section) and visualized by plotting corresponding polarization ellipses (see Fig. 1(c,d)). To increase visibility, the polarization ellipses are not drawn for every but for every fifth pixel and plotted on top of the measured intensity distribution (depicted in gray scale). Right-handed, left-handed and linear polarization states are illustrated in red, blue, and green, respectively, as indicated in Fig. 1(b). Experimental results show clearly separated areas of different polarization states as intended by the applied holograms (Fig. 1(j,p)). Remarkably, measured polarization states are not only resolved in their orientation *ψ* (s. Fig. 1(i,o)) but also in their ellipticity *ε* = tan(*χ*) (s. Fig. 1(h,n)) defined by the ratio of semi-major and semi-minor axes of the polarization ellipse. In both examples, all states of polarization are chosen to be part of a ring-like subspace of the Poincaré sphere (compare details of DPMS in method section) illustrated in Fig. 1(b), which means S_1_ = 0 while S_2,3_ ∈ [−1, 1]. This is also verified by the experimental results given in Fig. 1(e–g,k–m). Corresponding side-by-side holograms for the modulation of higher order Laguerre-Gaussian beams with structured polarization are shown in Fig. 1(j,p).

The shown LG-Poincaré beams have not been constructed by aligned superposition of two separately generated orthogonal modes but by direct modulation enabled by a liquid crystal display. Therefore, the spatial distribution of phase, amplitude, and polarization values becomes almost independent and gives a new degree of freedom to tailor complex light fields, i.e. it becomes possible to determine spatial distributions of polarization states within higher order light modes. In addition an auto-alignment is given due to direct polarization modulation by the DPMS. This opportunity can even be used to manipulate secondary parameters connected to certain spatial phase or polarization distributions as the optical angular momentum of light. In the following section we demonstrate such a case and show the new dimension of singular light patterns.

### Spatial modulation of spin and orbital angular momentum

**Figure 2:**
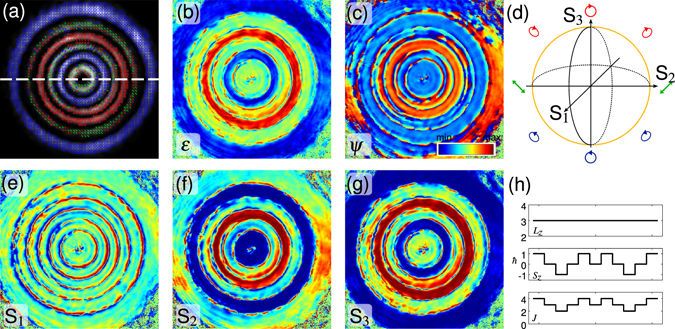
Spatial polarization modulation of a helical LG_5,3_ beam: (**a**) Polarization ellipses based on measured Stokes parameters S_1,2,3_ ∈ [−1, 1] shown in (**e**–**g**). Ellipticity *ε* ∈ [−1, 1] and orientation *ψ* ∈ [−*π*/2, *π*/2] of polarization states are given in (**b**) and (**c**). (**d**) Sketch of employed subspace of DPMS. (**h**) Distribution of spin (*S*_*z*_), orbital (*L*_*z*_) and total (*J*) angular momentum per photon along the white dashed line in (**a**).

Independent modulation of phase and polarization allows for spatial structuring of angular momentum carrying beams given by combination of spin (SAM) and orbital (OAM) angular momentum. We explore this opportunity by investigation of a higher order helical LG beam, which is visualized in Fig. 2. Due to its phase structure, the helical LG_5,3_ beam carries orbital angular momentum (OAM, ${\overrightarrow{L}}_{z}$) proportional to 3*ħ* per photon^30^. In addition, we introduce a spatially varying spin angular momentum (SAM, ${\overrightarrow{S}}_{z}$) by spatial polarization modulation of linear (${\overrightarrow{S}}_{z}=0$), right- (${\overrightarrow{S}}_{z}=-\hslash$) and left- (${\overrightarrow{S}}_{z}=+\hslash$) circular polarization states. The handedness of the circular polarization can be seen in Fig. 2(a,b) where measured polarization ellipses and the corresponding ellipticity (*ε*) are shown. Therefore the total amount of angular momentum $\overrightarrow{J}={\overrightarrow{L}}_{z}+{\overrightarrow{S}}_{z}$ is spatially modulated as indicated in Fig. 2(h) showing a cross-section of the beam along the white dashed line in Fig. 2(a). High quality of polarization modulation can also be seen from the orientation *ψ* of polarization states (Fig. 2(c)) as well as the three Stokes parameters (Fig. 2(e–g)).

The given example demonstrates the capability to tailor optical angular momentum structures by spatial phase and polarization modulation. Beside a general interest of singular optics to use this opportunity to investigate fundamental properties of such light fields, especially domains sensitive to optical angular momentum will profit from our investigations, as quantum communication and optical tweezers and its applications in optofluidics and biophotonics^31^.

### Holographic modulation of polarization singular structures

**Figure 3:**
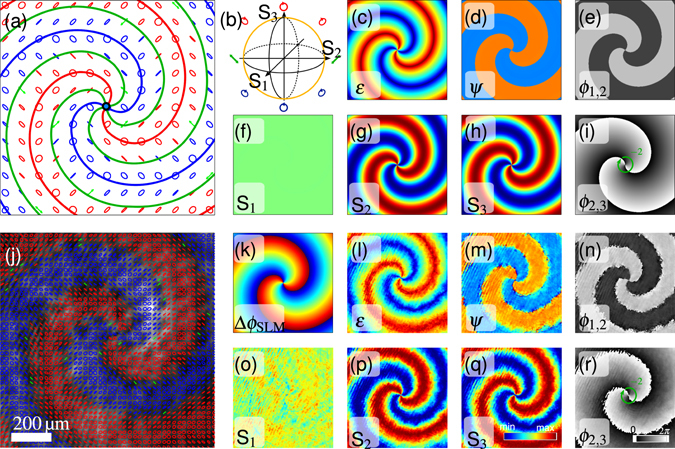
Polarization spiral containing various polarization singularities^34^: The polarization modulation hologram Δ*ϕ*_SLM_ in (**k**) results in a polarization field shown in ((**a**) simulation) and ((**j**) measurement). Appearing polarization singularities are sketched in (**a**) by green (L-line), red (right-handed C-line) and blue (left-handed C-line) lines and a black dot (V-point). The V-point is characterized *σ*_*P*_ = −2 estimated from the phase *ϕ*_2,3_ of the Poincaré field in (**i**) and (**r**). (**a**–**i**,**k**) show simulations while (**j**,**l**–**r**) give experimental results of Stokes parameters S_1,2,3_, ellipticity and orientation of the polarization ellipses (*ε*, *ψ*), as well as phases of complex Stokes fields *ϕ*_1,2_, *ϕ*_2,3_.

Holographic polarization modulation not only allows for spatial variation of different polarization states, but also gives the opportunity to create certain polarization structures which reveal a continuous polarization flow field including polarization singularities^4, 32, 33^. In Fig. 3 a polarization spiral embedding a vector singularity (V-point, i.e. point of undefined polarization) in its center is realized^34^. It is surrounded by intertwining lines of linear and left- or right-handed circular polarizations, so called L- and C-line singularities. In case of L- or C-line singularities the handedness or orientation of polarization ellipses is undefined, respectively. In between two adjacent line singularities elliptical polarization states form a smooth transition required for a continuous polarization flow field. To distinguish between different polarization singularities, they are sketched by green (L-line), red (right-handed C-line) and blue (left-handed C-line) lines and a black dot (V-point) in Fig. 3(a). To realize the polarization spiral experimentally, the DPMS is set to create polarization states on the meridian of the Poincaré sphere of the S_2,3_-plane (illustrated in Fig. 3(b)) by choosing orientations *θ*_1_ = −45° and *θ*_2_ = 90° for the first and second quarter wave plate, respectively (comp. method section). Experimental results of the spiraling polarization field resulting from the phase hologram Δ*ϕ*_SLM_ in Fig. 3(k) are given in Fig. 3(j,l–r) and can be compared to theoretical simulations shown in Fig. 3(a–i). The V-point is characterized by a conserved topological quantum number^32^, namely the Poincaré field index ${\sigma }_{P}=\frac{1}{2\pi }{\oint }_{C}d{\varphi }_{\mathrm{2,3}}=-2$ estimated (counterclockwise) around the V-point from the phase *ϕ*_2,3_ = arctan(S_3_/S_2_) of the Poincaré field shown in Fig. 3(i) and (r). In addition the phase *ϕ*_1,2_ = arctan(S_2_/S_1_) is shown in Fig. 3(e) and (n). Continuity of polarization parameters can be well seen from ellipticity of the polarization distribution (Fig. 3(c,l)) as well as the Stokes parameters (Fig. 3(f–h,o–q)). High experimental reproducibility of simulated singular structures generated by the DPMS is given in all three coordinates of the Poincaré sphere (i.e. Stokes parameter S_1_, S_2_, S_3_) as well as ellipticity and orientation of polarization ellipses (*ε*, *ψ*).

The direct modulation of spatial structures of polarization states as demonstrated in this example, allows to study complex Poincaré beams and polarization singular light fields. Auto-alignment of polarization components enables high modulation quality and enhanced stability compared to interferometric methods. This facilitates good comparability of experimental results and simulations. In particular, complex polarization singularity structures^35^ become realizable. For example, higher order singularities in form of polarization flowers and webs as well as hybrid polarization structures can be generated^33, 36^. In addition, beside transverse appearance and characteristics, especially dynamics of complex light fields, i.e. their longitudinal propagation, reveal a broad range of interesting findings as the evolution of phase and polarization singularities. As already phase as well as polarization singularities reveal interesting evolutions if considered independently, we expect distinctive dynamics if evolving polarization singularities are combined with naturally unfolding phase singularities. Dynamics provoked by customized light fields, generated by our dynamic holographic method, will be investigated in future.

## Methods

Any polarization can be defined by two orthogonal states, e.g. horizontal and vertical linear polarization, and is characterized by its amplitude ratio *A* ^÷^ = *A*_*v*_/*A*_*h*_ and phase shift Δ*ϕ* = *ϕ*_*v*_ − *ϕ*_*h*_ between these two components. A polarization sensitive device which only affects one of both components can be used to alter *A* ^÷^ and/or Δ*ϕ* and therefore change the polarization of light. For this purpose, nowadays liquid crystal spatial light modulators (SLM) are the device of choice as they allow for a computer controlled and spatially resolved (pixelwise) variation of the complex field^27^. While the polarization component parallel to the liquid crystal director (*P*_||_) is shifted in amplitude and phase, its orthogonal component (*P*_⊥_) is not altered for a phase-only and parallel aligned nematic-liquid-crystal SLM as used here^29, 33, 34^. The relative amplitude and phase also depend on the input polarization (${\overrightarrow{P}}_{{\rm{in}}}$). Accordingly, a dynamic polarization modulation system (DPMS) should consist of a SLM enclosed by two rotatable wave plates (WP) as explained in the following.

### Dynamic polarization modulation system – DPMS

**Figure 4:**
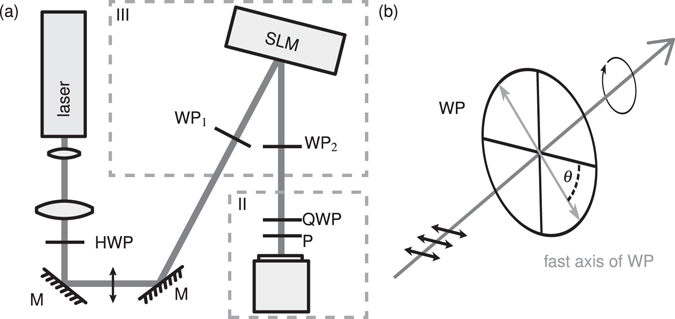
Schematic of (**a**) an experimental setup including (III) the DPMS and (II) a polarization measurement system; (**b**) the orientation angle *θ* of a wave plate (WP) defined as the inclination of its fast axis to the linear horizontal input polarization, here shown for *θ* = −45°. C: camera, (H/Q)WP: (half/quarter) wave plate, M: mirror, P: polarizer.

A rotatable wave plate is able to transform linear polarized light into elliptical or circular polarization states in dependence of the orientation angle *θ* of the fast axis of the wave plate with respect to the initial polarization. In our experimental realization depicted in Fig. 4 the first rotatable wave plate WP_1_ sets an initial amplitude ratio ${A}_{W{P}_{1}}^{\div}={A}_{\perp }/{A}_{\parallel }$ of *P*_⊥_ and *P*_||_ and an initial phase shift ${\rm{\Delta }}{\varphi }_{W{P}_{1}}$ of the light impinging on the SLM. Thus, the respective orientation angle *θ*_1_ defines the size of a subspace of states of polarization accessible by the SLM. Subsequently, the second rotatable wave plate WP_2_ allows to choose a set of polarization states determined by its orientation angle *θ*_2_ to finally yield the subspace of the whole DPMS being accessible at once, as explained later on. Summing up, the parameter of the DPMS are a spatially resolved amplitude factor *A*_SLM_(*x*, *y*) to influence the amplitude ratio *A* ^÷^, a spatially resolved phase shift Δ*ϕ*_SLM_(*x*, *y*) (both induced by the SLM), retardations of the two wave plates Γ_1,2_(*n*) = *λ*/*n* with ($n\in {\mathbb{N}}$) and their orientations *θ*_1,2_ of the fast axis. Figure 4(a) shows the setup including the DPMS (III) and a polarization measurement system (II) which is described later on. The orientation *θ* is defined as the angle between the input polarization and the fast axis of the WP (counterclockwise) in the direction of propagation. Exemplary, an orientation angle of *θ* = −45° is depicted in Fig. 4(b).

#### Mathematical description of DPMS by Jones formalism

For a comprehensive analysis of accessible subspaces and influence of parameters, the resulting states of polarization are represented on a Poincaré sphere which is a unit sphere spanned by the Stokes parameters (S_1_, S_2_, S_3_) with all fully-polarized states located on its surface. Linear polarization states can be found on the equator, while right circular and left circular polarization states mark the north and south pole, respectively. All possible output polarizations ${\overrightarrow{P}}_{{\rm{out}}}$ (=subspace) can be calculated with the Jones formalism to be

1 $${\overrightarrow{P}}_{{\rm{out}}}={M}_{{\rm{WP}}}({n}_{2},{\theta }_{2}){M}_{{\rm{SLM}}}({\rm{\Delta }}{\varphi }_{{\rm{SLM}}},{A}_{{\rm{SLM}}}){M}_{{\rm{WP}}}({n}_{1},{\theta }_{1}){\overrightarrow{P}}_{{\rm{in}}},$$

where the matrices of the reflective SLM and the two rotatable wave plates are given by

$$\begin{array}{rcl}{M}_{{\rm{WP}}}(n,\theta ) & = & (\begin{array}{cc}\cos (\theta ) & -\,\sin (\theta )\\ \sin (\theta ) & \cos (\theta )\end{array})\times (\begin{array}{cc}\exp (-i\frac{\pi }{n}) & 0\\ 0 & \exp (i\frac{\pi }{n})\end{array})\\  &  & \times (\begin{array}{cc}\cos (\theta ) & \sin (\theta )\\ -\,\sin (\theta ) & \cos (\theta )\end{array}),\\ {M}_{{\rm{SLM}}}({\rm{\Delta }}{\varphi }_{{\rm{SLM}}},{A}_{{\rm{SLM}}}) & = & (\begin{array}{cc}-{A}_{{\rm{SLM}}}(x,y)\exp (-i{\rm{\Delta }}{\varphi }_{{\rm{SLM}}}(x,y)) & 0\\ 0 & 1\end{array})\mathrm{.}\end{array}$$

#### Spatial phase retardation of polarization components

**Figure 5:**
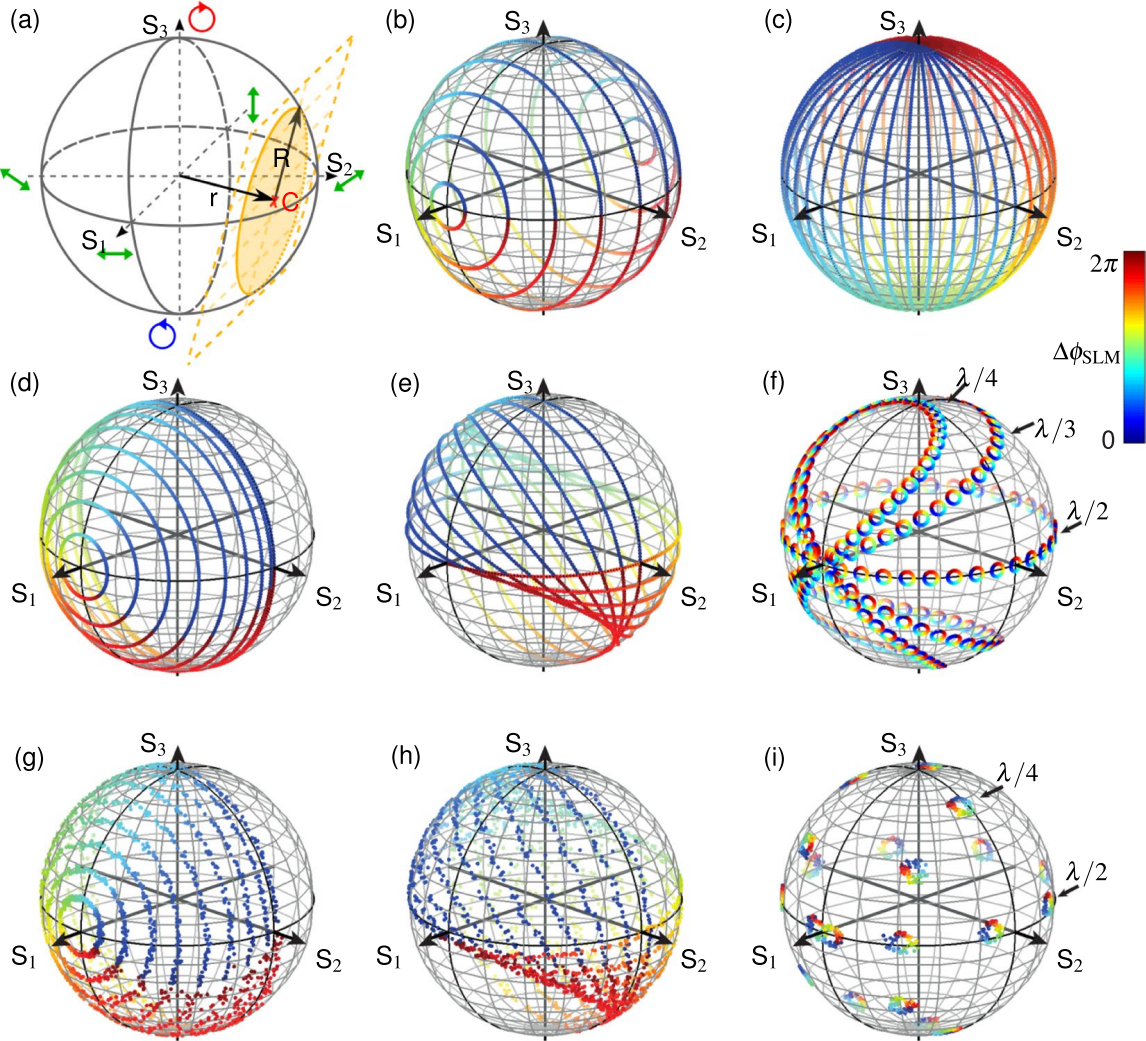
Dynamic polarization modulation: (**a**) Subspace of a DPMS. (**b**–**f**) Subspaces in dependence of WP_1_, influencing the radius *R* of the circle. Position of *C* depends on WP_2_ as investigated for different parameters in (**e**–**f**). Experimental results are shown in (**g**–**i**). Parameters for all sub-figures can be found in Table 1.

We use this formalism to evaluate various combinations of parameters and map each corresponding subspace of polarization states in dependence of the phase shift Δ*ϕ*_SLM_ of the SLM on the Poincaré sphere, as shown in Fig. 5. If the amplitude ratio *A* ^÷^ is not effected by the SLM (*A*_SLM_(*x*, *y*) = 1) the subspace of the DPMS can be described as the intersection of a plane and the surface of the Poincaré sphere which gives a circle in a three dimensional space, as illustrated in Fig. 5(a). This circle is uniquely describable by the vector $\overrightarrow{r}$(*r*, *ϑ*, *φ*) = (*r*(*θ*_1_), *ϑ*(*θ*_2_), *φ*(*θ*_2_)) from the origin of the sphere to the center *C* of the circle in spherical coordinates and its radius *R*(*θ*_1_). In many applications a 1:1 ratio of *P*_||_ and *P*_⊥_ (i.e. ${A}_{{{\rm{WP}}}_{1}}^{\div}=1$) is preferred which is facilitated by a retardation of *n*_1_ ∈ {2, 4} of WP_1_. Furthermore a quarter wave plate (*n*_1_ = 4) used as WP_1_ shows lower angle sensitivity which simplifies its handling in an experimental setup. In Fig. 5(b,d) several subspaces in dependence of *θ*_1_ are shown for a combination of two half and two quarter wave plates (used for WP_1,2_), respectively. Although in both cases WP_1_ is rotated about 45°, the circles are distributed over the whole Poincaré sphere for the half wave plates, while for the quarter wave plates only one half is covered, resulting in lower aberrations per angle uncertainty. In general, it is possible to access every polarization state by at least one combination of wave plates. An overview of all parameters used in Fig. 5 can be found in Table 1.

**Table 1:** Retardation Γ(*n*) = *λ*/*n* and orientation angle *θ*_1,2_ parameters of the two wave plates WP_1,2_ of simulations and experiments shown in Fig. 5.

Fig. 5	n_1_	*θ*_1_ (deg) from:step:to	n_2_	*θ*_2_(deg) from:step:to
(b)	2	2.5:5:45	2	90
(c)	4	−45	2	2.5:2.5:45
(d)	4	−90:−5:−135	4	90
(e)	4	45	4	45:5:90
(f)	4	−1	2, 3, 4	0:2.5:180
(g)	4	−90:−5:−135	4	90
(h)	4	45	4	45:5:90
(i)	4	−1.5	2	0:10:90
(i)	4	−1.5	4	15:15:165

To shift the subspace on the surface of the Poincaré sphere the second wave plate (WP_2_) is used. Its retardation determines the trajectory of $\overrightarrow{r}$ which follows a characteristic curve depending on *n*_2_ and *θ*_2_. For a half wave plate used as WP_2_ this trajectory is identical with the equator. Comparable to the longitudes of a sphere, Fig. 5(c) represents several subspaces of radius *R* = 1 (*θ*_1_ = 45°) for a variation of *θ*_2_ in case of WP_2_ being a half wave plate. If WP_2_ is replaced by a quarter wave plate, $\overrightarrow{r}$ follows a lemniscate trajectory (i.e. figure of eight) projected on the surface of the sphere and centered around the input polarization. Thus replacing the half wave plate used as WP_2_ enables to shift $\overrightarrow{r}$ of the subspace to elliptical and even circular polarization states and the subspace is no longer restricted to linear polarization states. For *R* = 1 a rotation of WP_2_ (=quarter wave plate) leads to a variable inclination of the circle’s plane with the equatorial plane, as presented in (e). As polarization states can be part of several circles, the flexibility of the DPMS is very high to choose between different combinations of parameters to spatially control the state of polarization of a light field. Especially a variable wave plate used as WP_2_ allows shifting $\overrightarrow{r}$ to nearly arbitrary positions on the Poincaré sphere, as illustrated in (f), where *θ*_1_ = −1° while *θ*_2_ is varied for *n*_2_ = {2, 3, 4}. Advantages of our setup compared to previous methods are the two independently rotatable wave plates which allow to tilt a subspace (circle) around the Poincareé sphere (comp. Fig. 5) and gain higher flexibility. Further, polarization control by a single SLM and auto-alignment of polarization components are benefits of our method.

Sub- Fig. 5(g–i) show the experimental results of Stokes parameter measurements of polarization states modulated with the DPMS. In the first example (g) WP_1_ is rotated in −5° steps by 45° while WP_2_ is fixed in analogy to the simulation in (c). It can be seen, that our measurements match theoretical predictions very well. In (h) WP_2_ is varied while WP_1_ is fixed. Here some deviations can bee seen which can be explained by an aberration of the retardation of the wave plate (within the retardance accuracy of *λ*/200 of the multi-order wave plate). This is verified by the measurement in (i) where $\overrightarrow{r}$ does not exactly match the S_3_ axis for *θ*_2_ = ±45° as expected for WP_2_ being a quarter wave plate, as well. As the retardation of a wave plate typically cannot be influenced, we recommend to use high quality wave plates, only.

#### Setting a spatial amplitude ratio of polarization components

In general, any state of polarization is describable as combination of a linear horizontal and a linear vertical polarization state and is explicitly defined by their phase shift Δ*ϕ* = *ϕ*_*v*_ − *ϕ*_*h*_ and amplitude ratio *A* ^÷^ = *A*_*v*_/*A*_*h*_. Both parameters can be influenced homogeneously for the whole beam profile by setting the orientation of the WPs. In addition, the SLM allows for a spatially resolved modification of phase shift of polarization components by introducing Δ*ϕ*_SLM_(*x*, *y*) as discussed in the previous section. By this method already a plethora of polarization structures can be realized as for example all possible combinations of linear polarizations, i.e. vector beams. However, it is desired to influence the amplitude ratio spatially as well, giving access to almost any polarization structure.

Therefore, we upgrade our method to not only allow polarization states represented on single rings on the Poincaré sphere but to enable polarization structures formed by polarization states given by continuous segments of the surface of the Poincaré sphere. For this purpose, spatial modulation of the amplitude ratio *A* ^÷^ of perpendicular polarization components is implemented. We developed a new method, which is related to combined amplitude and phase modulation^27^, but now applied to polarization modulation. More specific, spatially resolved variation of phase Δ*ϕ* and amplitude *A* ^÷^ ratio of polarization components is realized by a phase-only SLM. Therefore, the before mentioned phase Δ*ϕ*_SLM_(*x*, *y*) is accompanied by an additional amplitude factor ${A}_{{\rm{SLM}}}^{^{\prime} }(x,y)$.

The idea is to employ a blazed grating *φ*_*B*_ to diffract portions of one polarization component to a higher diffraction order (i.e. off-axis propagation) to affect the amplitude ratio *A* ^÷^ of modulated and non-modulated polarization components *P*_⊥_ and *P*_||_ within the zeroth order.

The zeroth order transmission function of the phase-only function $f(x,y)={e}^{i{A}_{{\rm{SLM}}}^{^{\prime} }{\phi }_{B}}$ is given by

2 $${T}_{{0}^{th}}(x,y)={e}^{i{A}_{{\rm{SLM}}}^{^{\prime} }(x,y)\pi }\cdot {\rm{sinc}}(-{A}_{{\rm{SLM}}}^{^{\prime} }(x,y\mathrm{)).}$$

**Figure 6:**
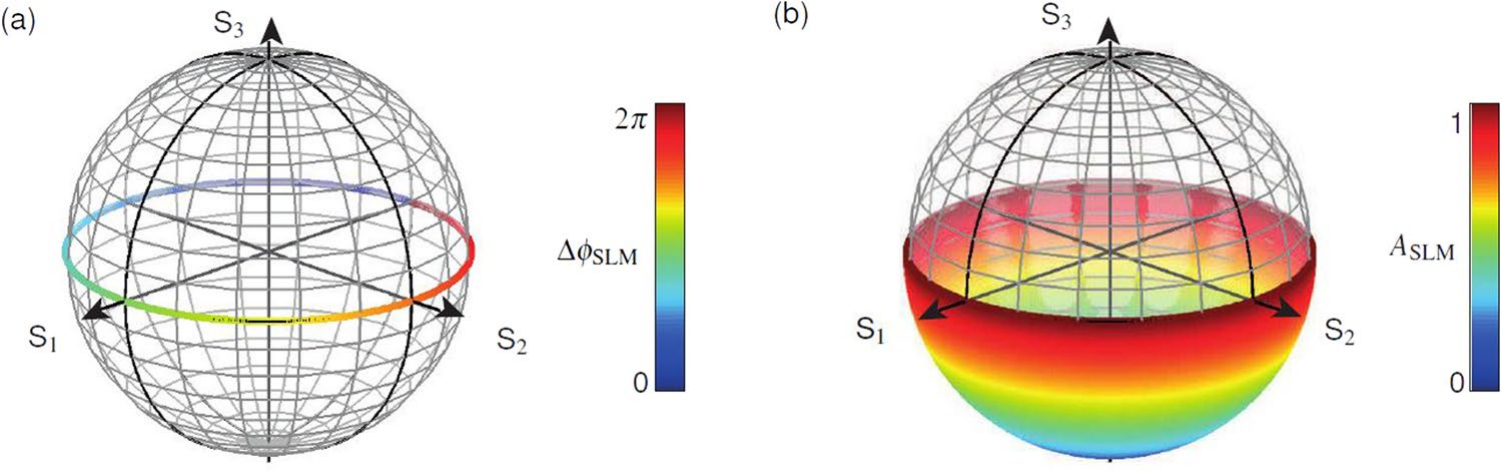
Spatial amplitude retardation of polarization components: Subspace of DPMS with (**a**) phase retardation only, and (**b**) phase retardation and additional influence to the amplitude ratio of polarization components (*θ*_1,2_ = −45°).

If we choose ${A}_{{\rm{SLM}}}={\rm{sinc}}(-{A}_{{\rm{SLM}}}^{^{\prime} })$ with *A*_SLM_, ${A}_{{\rm{SLM}}}^{^{\prime} }\in \mathrm{[0,}\,\mathrm{1]}$ Equation (2) employs the desired amplitude factor *A*_SLM_. To eliminate the additional phase term, one has to multiply by its complex conjugate. Employed within the DPMS this technique directly effects the amplitude ratio

$${A}_{{\rm{DPMS}}}^{\div}(x,y)={A}_{{{\rm{WP}}}_{1}}^{\div}\cdot \frac{1}{{A}_{{\rm{SLM}}}(x,y)}\cdot {A}_{{{\rm{WP}}}_{2}}^{\div}$$

of the polarization components and can be combined with the modulation of the phase shift

$${\rm{\Delta }}{\varphi }_{{\rm{DPMS}}}(x,y)={\rm{\Delta }}{\varphi }_{{{\rm{WP}}}_{1}}+{\rm{\Delta }}{\varphi }_{{\rm{SLM}}}(x,y)+{\rm{\Delta }}{\varphi }_{{{\rm{WP}}}_{2}}$$

between polarization components as discussed in the previous section. In Fig. 6 a comparison of subspaces of the DPMS is shown (a) with phase retardation only, and (b) with simultaneous modulation of phase retardation and amplitude ratio. It can be clearly seen, that the subspace which also effects the amplitude ratio covers a continuous segment of the surface of the Poincaré sphere and is not limited to a single ring. The orientation and size of the surface depends on Γ_1,2_(*n*), *θ*_1,2_, Δ*ϕ*_SLM_(*x*, *y*) and *A*_SLM_(*x*, *y*).

### Spatially resolved Stokes parameter measurements

In the experiment a reflective SLM is illuminated under a small angle by a linearly polarized laser (*λ* = 532 nm). A half wave plate (HWP in Fig. 4(a)) allows to choose a linear horizontal input polarization which is parallel to the director of the liquid crystal display and a polarization measurement system depicted in Fig. 4(a) (II). To evaluate the polarization modulation of the DPMS, intensity (*I*) measurements behind a rotating quarter wave plate (rotation angle *θ*_3_) and a fixed linear polarizer are related to the corresponding Stokes vector of the polarization state^22^ by Fourier analysis:

3a $${S}_{0}=\frac{2}{N}\sum _{j=1}^{N}{I}_{j}-2{I}_{j}\,\cos (4{\theta }_{{3}_{j}})$$

3b $${S}_{1}=\frac{8}{N}\sum _{j=1}^{N}{I}_{j}\,\cos (4{\theta }_{{3}_{j}})$$

3c $${S}_{2}=\frac{8}{N}\sum _{j=1}^{N}{I}_{j}\,\sin (4{\theta }_{{3}_{j}})$$

3d $${S}_{3}=\frac{4}{N}\sum _{j=1}^{N}{I}_{j}\,\sin (2{\theta }_{{3}_{j}}),$$

where *N* > 8 is the number of measurements (minimum given by sampling theorem). To yield normalized Stokes parameters *S*_1,2,3_ have to be divided by *S*_0_. Within the experimental setup, we employed a camera to enable spatially resolved Stokes parameter measurements. While for homogenous polarization distributions it is possible to take average values of several camera pixels, for the complex spatially structured Poincaré beams a pixel wise evaluation of Stokes parameters is performed. Opposite to other methods frequently used for vector beam analysis, as e.g. intensity observations behind a rotating polarizer, here beside orientation also ellipticity information of polarization states is recorded.

## Conclusion

Simultaneous modulation of spatial phase, amplitude, and polarization values by a holographic method enables to tailor complex Poincaré beams, advanced optical angular momentum structures and various kinds of polarization singularities. To show the potential of the method, we demonstrate the generation of higher order LG modes combined with spatial modulation of polarization states. However, our holographic modulation system (DPMS) is not restricted to LG beams, but allows generating any light mode together with linear, elliptic and circular states of polarization in various combinations. Furthermore, spatial combination of spin and orbital angular momentum, as shown for a higher order helical LG beam, can be employed in quantum communication and classical entanglement^37^. Moreover, such polarization tailored light beams are of interest for high NA focusing and its applications, where three-dimensional polarization structures including z-polarization components can be tailored^38^. Of special interest are experimental results and simulations of complex Poincaré beams embedding different types of polarization singularities. Realization of polarization singularities (V-points, L-lines and C-lines) and their analysis are of general importance as singular structures are not only present in optics but (among others) also occur in astronomy or Bloch walls of magnetic systems. Although already several polarization singularities have been modulated with the DPMS, our system will lead to experimental realizations of even more complex polarization structures^32, 33, 35, 36^ combining several types of singularities to whole polarization networks. Additionally, we expect distinctive dynamics if (unstable) polarization singularities are combined with (unfolding) phase singularities. Finally, capabilities of the DPMS have been evaluated within the method section. It has been characterized with respect to the phase of the SLM, the retardation of the wave plates and their orientation to demonstrate the enormous flexibility of the method.
